# Preparations of Poly(lactic acid) Dispersions in Water for Coating Applications

**DOI:** 10.3390/polym13162767

**Published:** 2021-08-18

**Authors:** Giada Belletti, Sara Buoso, Lucia Ricci, Alejandro Guillem-Ortiz, Alejandro Aragón-Gutiérrez, Olga Bortolini, Monica Bertoldo

**Affiliations:** 1 Department of Chemical, Pharmaceutical and Agricultural Sciences, University of Ferrara, Via L. Borsari 46, 44121 Ferrara, Italy; giada.belletti@unife.it (G.B.); olga.bortolini@unife.it (O.B.); 2 Institute of Organic Synthesis and Photoreactivity, National Research Council, Via P. Gobetti 101, 40129 Bologna, Italy; sara.buoso@isof.cnr.it; 3 Institute for Chemical and Physical Processes, National Research Council, Via G. Moruzzi 1, 54124 Pisa, Italy; lucia.ricci@pi.ipcf.cnr.it; 4 Instituto Tecnológico del Embalaje, Transporte y Logística, ITENE, Calle de Albert Einstein 1, 46980 Paterna, Spain; alejandro.guillem@itene.com (A.G.-O.); alejandro.aragon@itene.com (A.A.-G.)

**Keywords:** poly(lactic acid), water emulsions, water dispersions, film formation

## Abstract

A green, effective methodology for the preparation of water-based dispersions of poly(lactic acid) (PLA) for coating purposes is herein presented. The procedure consists of two steps: in the first one, an oil-in-water emulsion is obtained by mixing a solution of PLA in ethyl acetate with a water phase containing surfactant and stabilizer. Different homogenization methods as well as oil/water phase ratio, surfactant and stabilizer combinations were screened. In the second step, the quantitative evaporation of the organic provides water dispersions of PLA that are stable, at least, over several weeks at room temperature or at 4 °C. Particle size was in the 200–500 nm range, depending on the preparation conditions, as confirmed by scanning electron microscope (SEM) analysis. PLA was found not to suffer significant molecular weight degradation by gel permeation chromatography (GPC) analysis. Furthermore, two selected formulations with glass transition temperature (Tg) of 51 °C and 34 °C were tested for the preparation of PLA films by drying in PTFE capsules. In both cases, continuous films that are homogeneous by Fourier-transform infrared spectroscopy (FT-IR) and SEM observation were obtained only when drying was performed above 60 °C. The formulation with lower Tg results in films which are more flexible and transparent.

## 1. Introduction

In recent years, in an effort to produce more eco-friendly products, renewable and biodegradable polymers have been investigated as emerging coating materials. One of the most promising polymers for such a purpose is poly(lactic acid) (PLA) due to its biodegradability, biocompatibility and production on an industrial scale at relatively low cost [1,2,3]. PLA is already used for a wide range of applications, particularly in the packaging sector [4], for instance for making food and beverage containers, cups, overwrap, blister packages, as well as coating on paper and board [5,6,7]. The most frequently used conversion method for the preparation of PLA items is melt processing, such as cast extrusion, extrusion coating, lamination and blown extrusion [8,9]. An alternative technology for the preparation of coatings that does not involve treatment at high temperature is solvent casting [10,11]. The method requires the solubilization of the polymer in a suitable solvent, followed by casting of the solution onto the substrate. Despite the possibility to modulate the film thickness by varying the concentration of the polymer solution, the release of toxic and harmful organic solvents during the process gave rise to health and environmental issues. For this reason, the latter method is not typically used to produce biodegradable films. Indeed, in the last decades, many efforts have been devoted to the replacement of solvent-borne polymer coatings with their waterborne counterparts, even for those applications where they were traditionally used such as adhesives and inks [12,13]. Water, indeed, is considered as a cheap, safe, non-toxic and environmentally benign solvent [14]; however, polymers are rarely soluble in water. A typical method to use water as a vehicle, in spite of the absence of solubility, is the preparation of polymer latexes consisting of polyacrylate, polymethacrylate and more recently, polyurethane as well [15,16,17,18,19,20,21,22]. Water dispersions are gaining land in many application fields such as paints [23], adhesives [24] and inks [25,26], particularly for the food packaging sector [27,28], where the restrictions on the possible substances used forced the producers to substitute old technologies with safer ones.

When water dispersions (or latexes) are used to prepare coatings, the preparation is cast onto the chosen substrate and, as water evaporates, the polymer particles merge and form a homogenous film on the coated surface [29]. The ability of a polymer latex to form a continuous film depends on the minimum film formation temperature (MFFT) of the polymer. When the casting takes place above the MFFT, the particles undergo deformation and cohesion, thus creating a homogeneous film. On the other hand, when the film is cast below its MFFT, a brittle discontinuous film or compact powder may be obtained [30]. Typically, the MFFT tends to be close to the glass transition temperature (Tg) of the polymer or above it [31]. The most commonly accepted mechanism of film formation from waterborne dispersions of preformed polymers is composed of three stages: drying, deformation and coalescence [32,33]. During drying, the water evaporates from the dispersion surface at a constant rate until the polymer has reached 60–70% of volume fraction. As the water evaporation rate slows down, deformation occurs and the particles come into irreversible contact. In the final step, polymer chain interdiffusion takes place and the particles coalesce into a continuous film.

The most common methods to produce water-based coatings are emulsion polymerization [34] and emulsification of preformed polymers. Polymer emulsions of acrylate and methacrylate have been produced for many years with emulsion polymerization [34,35,36,37,38]. More recently, water dispersion of low molecular weight resins that are converted into high weight mechanically performing coatings by UV-curing have also entered into the market [39,40]. Unluckily, emulsion polymerization is applicable only to free radical polymerizable monomers and lactide and lactic acid are not included among them. Indeed, in these cases the production of water-based preparations is achieved by dispersing the preformed polymer [41]. For instance, in the acetone process for the preparation of polyurethane-polyurea water dispersions, the hydrophilic isocyanate prepolymer is firstly chain extended in acetone to give the desired molecular weight. Subsequently, by the addition of water and removal of the solvent, a purely waterborne dispersion is formed [42,43].

To the best of our knowledge the preparation of aqueous emulsions and dispersions of PLA has been reported only for applications in the biomedical field, especially for the obtainment of PLA nanoparticles for drug delivery [44,45]. Some of the methods used in these cases involve the use of non-harmful solvents, such as acetone and ethyl acetate. Very diluted dispersions of PLA particles have been obtained by nanoprecipitation [46,47,48,49], salting-out [41,50,51,52] and emulsification-diffusion methods [46,53,54]. Nevertheless, these works aim at the isolation through centrifugation or lyophilization of the PLA nanoparticles. As a consequence, the dispersion stability over time is usually not investigated and besides the final PLA content is usually lower than 5 wt.%. On the other hand, with the use of chlorinated solvents by emulsification-evaporation methods, PLA dispersions with higher polymer contents are accessible (maximum 20 wt.%) [27,55,56,57]. Generally, the simplest emulsion-based procedures require the solubilization of PLA in a water-immiscible organic solvent, followed by subsequent dispersion of this organic phase in fine droplets into the aqueous medium. After that, the organic solvent evaporates leading to the formation of PLA nanoparticles dispersed in water [44]. This technique was also exploited to prepare blends of PLA with hydrophilic polymers such as chitosan [58,59], lignin [60] and nanocellulose [61,62]. However, in the latter cases, the studies focus on the preparation of thermoplastic composites and consequently, the stability of the dispersion was not an issue and thus it was not reported. Besides, the toxicity associated with the chlorinated solvents used in these processes limits the biocompatibility and the use of the dispersions thus prepared for food contact applications. Additionally, the preparation of waterborne PLA dispersions for coating applications has been the subject of some recent patents [63,64]. In these cases, preparations with high dry content (25–70 wt.%) are afforded in the absence of organic solvent by continuously and simultaneously introducing melted PLA and aqueous phase into the blender of an emulsifying unit. Nevertheless, with these techniques the polymer is always processed at high temperatures and usually, high amounts of viscosity reducing agents, plasticizers or additives are needed. The PLA particles thus obtained are in the micrometric size range.

Aim of the present work was the design and optimization of a process to obtain stable dispersions of PLA in water suitable for coating film formation. The process had to be accomplished without chlorinated solvents to allow the use of the produced coatings in food packaging application. To this aim, an amorphous PLA soluble in non-chlorinated solvents and a solvent approved for food contact application, such as ethyl acetate, were selected. Different homogenization methods were tested for the mixing of the organic phase, in which PLA was dissolved, and the water-phase containing emulsifier and stabilizer. With the purpose of ensuring the stability of the preparation during storage and during evaporation of the organic solvent, the use of several emulsifiers and different polysaccharides as stabilizers were examined. In fact, to prepare kinetically stable emulsions, an emulsifier is needed to protect the newly formed droplets against the different destabilization mechanisms. In particular, the emulsifier forms a protective interfacial layer when it adsorbs on the surface of the droplets, preventing the latter from merging together [65]. Emulsifiers can be produced using either petrochemical or bio-based feedstocks and they are commonly classified according to the polarity of their head group: anionic, cationic, non-ionic and amphoteric [66]. In recent years, due to the continuous consumption and depletion of fossil fuels, renewable substrates have been of increasing interest for the production of bio-based surfactants [67]. On the other hand, non-surface-active polysaccharides, like gelatinized starch, may contribute to interfacial stabilization of an oil-in-water emulsion by interaction with the surfactant layer already located at the interface [65,68]. Additionally, polysaccharides are able to enhance the stability of emulsions by increasing the viscosity of the aqueous phase [69]. In this work, various chemicals and polysaccharides approved for food contact application were chosen as promising surfactants to be tested. Furthermore, several emulsifiers were selected on the basis of the relative strength of their hydrophilic and lipophilic moieties (known as hydrophilic–lipophilic balance or HLB) [70]. Surfactants with both high and low HLB values were examined. Regarding the choice of the stabilizers, the attention was focused on bio-based polysaccharides, which are largely employed in the food industry such as starch and xanthan gum (XG) [71,72] or promising emulsion stabilizers such as microfibrillated cellulose (MFC) [73]. Additionally, the film forming ability of two selected PLA dispersion formulations was studied at different temperatures in the 25–110 °C range. The morphology of the obtained layers was investigated through scanning electron microscopy (SEM) to identify the MFFT of the innovative biodegradable dispersions in water.

## 2. Materials and Methods

### 2.1. Materials

PLA Ingeo Biopolymer 4060D (PLA) was supplied by NatureWorks LLC (Blair, NE, USA.). Sodium dodecyl sulfate (SDS), ethyl acetate, sodium alginate (SA), sodium carboxymethylcellulose (NaCMC), Tween 80, Tween 20, stearoyl-2-lactylate (SSL) and Span80 were purchased from Sigma-Aldrich (Milan, Italy). Starch C*Icoat 07,525 (starch) and xanthan gum (XG) were generously supplied by Cargill (Krefeld, Germany,). A solution of starch at 3% in ultrapure water was prepared by heating under reflux 6 g in 200 mL for nearly 1 h. Metolad 368 (Met. 368), Metolad 388 (Met. 388), Leukonol LB-2 (Leu.) and Tafigel AP 15 (Taf.) were kindly provided by Münzing Chemie GmbH (Abstatt, Germany). Synperonic PE/F68 (SYN) was generously supplied by Croda Chemicals (Mortara (PV), Italy). Microfibrillated cellulose MFC Exilva F 01-L (MFC) was kindly supplied by Borregaard (Sarpsborg, Norway).

### 2.2. Preparation of PLA Dispersions with Sodium Dodecyl Sulfate as Surfactant and Starch as Stabilizer

6.47 g of starch solution at 3% conc. was diluted with 28.5 mL of ultrapure water (starch conc. 0.5 wt.%) and 0.28 g of SDS were added (SDS conc. 0.8 wt.%). In the meantime, 5 or 7 g of PLA was solubilized in 45 or 87.5 mL of ethyl acetate (EtOAc) (11 or 8 wt./vol.%, respectively) under vigorous stirring for 4 h at room temperature. After that, proper amounts of the oil and water phases were mixed, in an ice bath, with the use of a homogenizer to obtain the emulsions. Different tests were carried out by changing the volume ratio between the two phases as well as the total phase volume (entries 1–13, Table 1). For homogenization, ULTRA-TURRAX (UT) or two different ultrasound probes (UVC and UH) were tested. Homogenization conditions were as follows: (a) UH for 30 s at amplitude 50% + 1 min 30 s at amplitude 80%; (b) UVC for 30 s at amplitude 50% + 2 min of overhead stirrer at 200 rpm + (30 s at amplitude 90% + 2 min of overhead stirrer at 200 rpm) three times; (c) UT for 1 min 30 s at 1100 rpm. Afterwards, the resulting emulsions were stirred for 20 h at room temperature and 200 rpm under the aspiration of a laboratory hood (suction speed: 0.5 m/s).

A similar procedure was adopted in tests of entry 11 and 12 in Table 1 except that the amount of the starch solution used was 0.38 g (starch conc. 1.1 wt.%) in test 11 and the amount of SDS employed in test 12 was 0.10 g (SDS conc. 0.3 wt.%).

### 2.3. Preparation of PLA Dispersions with SDS and Different Polysaccharides as Stabilizers

Different amounts of starch, MFC or XG and 0.28 g of SDS were solubilized in 35 mL of ultrapure water (Table 2). In the case of MFC, the polysaccharide suspension was treated with UT at 10,000 rpm for 4 min before addition of SDS. In the meantime, 5 g of PLA was solubilized in 45 or 62.5 mL of EtOAc (11 or 8 wt./vol.%, respectively) under vigorous stirring for 4 h at room temperature. After that, the oil and water phases were mixed together, in an ice bath, with the aid of the UVC homogenizer for 30 s at amplitude 50% + 2 min of overhead stirrer at 200 rpm + (30 s at amplitude 90% + 2 min of overhead stirrer at 200 rpm) three times. Afterwards, the resulting emulsion was stirred for 20 h at room temperature at 200 rpm under the aspiration of a laboratory hood (suction speed: 0.5 m/s).

### 2.4. Preparation of PLA Dispersions with Different Surfactants Than Sodium Dodecyl Sulfate

The proper amount of emulsifier was solubilized into 35 mL of ultrapure water. In the case of the presence of starch as stabilizer in the formulation, the 35 mL included 6.47 g of starch solution at 3% conc. (starch conc. 0.5 wt.%). The type of surfactant employed and its concentration in the water phase are listed in Table 3.

Moreover, 5 g of PLA was dissolved in 45 mL of EtOAc under vigorous stirring for 4 h at room temperature. When Span 80 was employed as surfactant, the latter was solubilized in the organic phase together with PLA. After that, the oil and water phases were mixed together and homogenized while standing in an ice bath to obtain the emulsions. Homogenization was carried out with UH or UVC ultrasound homogenizer under conditions “a” or “b” mentioned in Section 2.2, respectively. Afterwards, the resulting emulsions were stirred overnight at 200 rpm under the aspiration of a laboratory hood (suction speed: 0.5 m/s).

### 2.5. Preparations of PLA Films from Dispersions

1.7–3.0 g of PLA dispersions prepared under the condition of entry 3, Table 1 (PLA_SDS_starch) or entry 4, Table 3 (PLA_SYN), were transferred into a PTFE capsule (diameter 3.7 cm) and allowed to dry for 2–3 h at the selected temperature until solidification occurred. The drying temperature was room temperature and then the latter was gradually increased by 10 °C starting from 40 up to 110 °C.

### 2.6. Instruments and Characterization Methods

To weigh the starting materials Entris Sartorius and Sartorius BP61S laboratory balances (Sartorius AG, Goettingen, Germany) were used. To prepare the water and the organic phase of the dispersions, an IKA magnetic stirrer RCT basic (IKA, Staufen, Germany) was employed. Ultrapure water was produced with Millipore Direct-Q 3UV (Merck KGaA, Burlington, MA, USA).

For homogenization of oil and water phases, Ultrasound Hielscher Up200St (UH) equipped with a 14 mm diameter probe, Vibra-Cell Ultrasonic Liquid Processors VCX750 (UVC) (Sonics, Newtown, MA, USA) provided with a 13 mm diameter probe and IKA ULTRA-TURRAX T 25 basic (UT) equipped with an S 25 N-18 G dispersing tool (IKA, Staufen, Germany) were used.

Emulsions and dispersions were stirred with an ArgoLab AM20-D (ArgoLab group, Landshut, Germany) overhead stirrer equipped with PTFE blade.

The diameter of the particles was determined by dynamic light scattering (DLS) analysis at 25 °C using a NanoBrook Omni particle size analyzer (Brookhaven Instruments Corporation, Holtsville, NY, USA) equipped with a 35-mW red diode laser (nominal 640 nm wavelength), BI-SCP cell in backscattering (173°) and software for data analysis. To give reliable results, dilute suspensions (0.15 vol./vol.%) were analyzed. For data elaboration, reflective indices of the dispersion medium and of the dispersed phase were assumed to be 1.330 and 1.596, respectively. Each measurement was repeated five times on the same sample and the reported data were the average over the five measurements. The standard deviation δ (Equation (1)) was assumed as data error, where N is the number of measurements, x_i_ the value of the i-th measurement and µ the arithmetic mean. All the DLS data were analyzed by a multimodal function. Particles were assumed to be uniform spheres.

(1) $$\delta =\overset{N}{\underset{i=1}{\sum }}\frac{\sqrt{{\left(x_{i}-\mu \right)}^{2}}}{\sqrt{N}}\;\;\;$$

The dispersions’ stability was assessed through visual criteria and DLS analysis both after the preparation and after 1 week. Dispersions were assumed stable if no phase separation was observed and the particle size variation was lower than the standard deviation on the particle size value.

The dry residuum of the preparations was determined after heating the sample at 200 °C for 30 min in an oven.

The residual amount of EtOAc present in the emulsion was quantitatively determined by gas chromatography/mass spectroscopy analysis (GC/MS). A triple quadrupole GC/MS instrument from Agilent Technologies (Agilent Technologies, Inc., Santa Clara, CA, USA) equipped with a 20 × 0.18 mm, 0.18 µm column was employed, using hydrogen at 0.8 mL/min as carrier gas with a 10:1 split ratio. For the analysis, 100 µL of the emulsion was added to a 20 mL chromatography vial and incubated at 85 °C for 30 min for head-space extraction. The GC oven was programmed for 40 °C for 3 min, followed by a stepped increase of 10 °C min^−1^ to 50 °C, where it was held for 5 min, and then the temperature was increased by 30 °C min^−1^ to 230 °C, where it was held for 3 min. The quantitative determination of EtOAc traces was conducted by using the selected ion monitoring (SIM) at the *m*/*z* values 88/73. Calibration was performed by injecting solutions of EtOAc in *N*,*N*-dimethylformamide at known concentrations into the GC/MS instrument.

Differential scanning calorimetric analyses were performed on a DSC 8000, PerkinElmer Inc. (Waltham, MA, USA) instrument equipped with IntraCooler II cooling device and Pyris software (Version 13.3, PerkinElmer Inc., Waltham, MA, USA) for instrument control, data acquisition and analysis. The instrument was calibrated for temperature and energy with high-purity indium and lead as standards. 3–10 mg of sample was analyzed in aluminum pans under dry nitrogen atmosphere (30 mL/min). Samples were at first heated up from 25 to 200 °C to erase the thermal history and to remove any trapped volatile substance such as residual solvents. Thus, samples were cooled down to −70 °C (cooling step), maintained at −70 °C for 5 min and finally heated up again to 200 °C (second heating step). Heating and cooling steps were all performed at 10 °C/min as the scanning rate.

Size-exclusion chromatography (SEC) analyses were performed with a Jasco (Jasco Europe srl, Cremella, Italy) instrument comprising a PU-2089 Plus quaternary pump and injector with a 20 mL loop, two in-series PLgel MIXED-D columns (Agilent Technologies Italia S.p.A., Cernusco sul Naviglio, Italy; linearity range 200 to 2,000,000 g/mol based on polystyrene equivalent) placed in a Jasco CO-2065 column oven set at 30 °C, a Jasco RI-2031 Plus refractive index detector, and a Jasco UV-2077 Plus multi-channel UV-Vis detector. The samples in the form of films or powders were dissolved in trichloromethane (HPLC grade Sigma-Aldrich, Milan, Italy) with the aid of sonication and filtered through a 0.2 mL pore size PTFE filter to remove the insoluble fraction before injection as 5 mg/mL solutions; elution was performed with trichloromethane at 1 mL/min flow rate. ChromNav Jasco software (Jasco Europe srl, Cremella, Italy) was used for data acquisition and analysis based on a calibration curve obtained by running a set of four monodisperse polystyrene standards (19,000, 50,000, 233,000, and 300,000 g/mol, respectively) and performing a 4th order fit.

Scanning electron microscopy analyses (SEM) were accomplished with a Zeiss EVO 40 microscopy (Carl Zeiss Microscopy Ltd., Cambridge, UK) equipped with a LaB_6_ source. Samples in the form of powder or film were gold sputtered before observation. Film sections were obtained by fracture in liquid nitrogen.

A Perkin Elmer Lambda 650 UV-Vis spectrophotometer (Waltham, MA, USA) was used to record the transmittance of free-standing films. Measurements were accomplished in transmission mode with air as reference. Film thickness was measured with an electronic outside micrometer that is able to perform measurement between 0.001 and 25 mm.

FT-IR spectra were recorded on an Agilent Cary 630 FTIR spectrophotometer (Agilent Technologies, Inc., Santa Clara, CA, USA) with ZnSe ATR element. Background and sample spectra were collected by accumulating 32 scans.

## 3. Results and Discussion

### 3.1. Influence of the Preparation Conditions on the Stability of PLA Dispersions in Water

**Figure 1:**
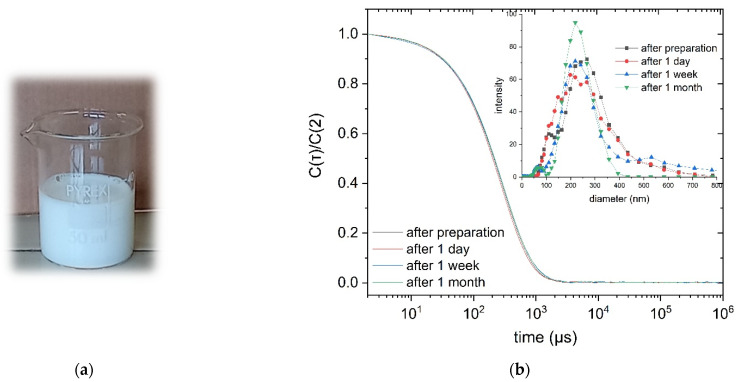
(**a**) Picture of the PLA dispersion of entry 5, Table 1; (**b**) comparison between DLS plots of the PLA dispersions prepared under the condition of entry 3, Table 1, after storing for increasing periods of time. Correlograms are normalized with respect to the intensity at time 2 μs. Insert refers to the particle size distribution as obtained by data fitting with a multimodal size distribution function.

Several tests to prepare ethyl acetate/water (EtOAc/H_2_O) emulsions of PLA with SDS as surfactant and starch as stabilizer were carried out (Table 1), by using Ultraturrax (UT) or Ultrasound processors (UH and UVC) as homogenizer. When Ultraturrax (UT) was used, the emulsion formed but it was unstable (entry 1, Table 1); during the subsequent evaporation of the organic solvent, the majority of the preparation coagulated. On the contrary, with the use of ultrasound processors, either Ultrasound Hielscher UP200St (UH) or Vibra-Cell Ultrasonic Liquid Processors VCX750–SONICS Materials (UVC), stable PLA emulsions in water were obtained (Figure 1a). As a matter of fact, by solvent evaporation, these were converted into stable dispersions with size of 200–215 nm. No significant effect of the specific ultrasound processor used on the particle size was noticed (Table 1). Dry matter content was between 13 and 20 wt.%, which is much higher than any other reported result for stable PLA dispersions in water produced with emulsification-evaporation methods and non-chlorinated solvents [29,55].

Inspired by the good results obtained with ultrasound homogenizers, we attempted to further increase the PLA content in water by using a larger volume of organic phase, namely, 62.5 mL instead of 45 mL, to prepare the emulsion with the UVC homogenizer (entry 4, Table 1). However, even if the emulsion formed, the process was not effective since a cloth made of PLA formed on the stirrer during the subsequent EtOAc evaporation. When the process was replicated with the same oil and water phase volumes, but with 8% concentration of PLA instead of 11% in the organic phase, once again a homogeneous emulsion was obtained.

In this case, the emulsion was stable enough to be converted into a stable dispersion by EtOAc evaporation. Particle size was smaller than the one obtained in the previous test with higher PLA concentration (entry 5, Table 1). Similar results were afforded when the total volume of the organic and water phases was reduced from 97 to 32 mL (entry 6, Table 1). The results suggest that the viscosity of the oil phase is the key parameter allowing the dispersion in the case of the higher oil/water phase ratio. Indeed, dispersion was achieved with PLA concentration of 11% and phase ratio of 1.3 or PLA concentration of 8% and phase ratio of 1.8. The decrease of the PLA concentration in EtOAc leads to a lower viscosity of the organic phase, thus reducing the energy input needed to disperse the organic phase into the aqueous one [74]; as a consequence, the treatment of a larger total volume becomes feasible. The further attempt to prepare a more concentrated PLA dispersion by increasing to 87.5 mL the volume of the 8% PLA solution (oil/water phase ratio of 2.5), once again gave an emulsion. However, this was not very stable and a clot formed on the stirrer during evaporation of the organic solvent, if the UVC homogenizer was used (entry 7, Table 1). On the contrary, with UH homogenizer, after solvent evaporation, the emulsion turned into a homogeneous dispersion with 20% dry matter content (entry 8, Table 1). Even when the preparation was performed in a smaller scale (with a lower total volume) but with the use of UVC homogenizer, a clot on the stirrer was formed during evaporation of the EtOAc (entry 9, Table 1). This result clearly indicates the higher efficiency of UH in transferring the energy and in treating larger both total volume and EtOAc/H_2_O volume ratio than UVC does. Indeed, UH sonication was effective in treating EtOAc/H_2_O volume ratio up to 2.5 (entries 2, 8, 10 and 11, Table 1), whilst the ratio was only up to 1.8 for preparations performed with UVC (entries 3, 4, 5, 6, 7 and 9, Table 1). In any case, the maximum EtOAc/H_2_O volume ratio that could be treated is 2.5. As a matter of fact, no stable preparation could be obtained with a ratio of 5, even with the UH homogenizer, a relatively low total volume (60 mL) and a high concentration of stabilizer, 1.1% instead of 0.5% (entry 11, Table 1).

When the ratio between phases was lower than 2.5 for treatment with UH and not higher than 1.8 for UVC use, stable emulsions and then dispersions were obtained, even with SDS concentration lower than 0.8%. Indeed, good results were obtained with SDS 0.3% (entry 12, Table 1) plus 0.5% of starch.

### 3.2. Influence of Ethyl Acetate Residua on the Emulsion Stability

The homogeneous emulsions obtained in the experiments described in Table 1, if left standing without stirring, coagulated in a reasonably short time. However, it was clear that they turned into stable dispersions after complete evaporation of the organic solvent (Figure 1a). Dispersions can even be stored at room temperature or in the fridge at +4 °C for days and months without sedimentation and significant change of the particles’ size and of their distribution (Figure 1b).

**Figure 2:**
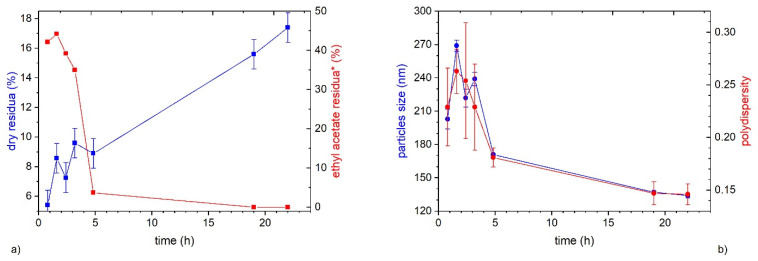
(**a**) Kinetics of ethyl acetate residue (red line) and dry residue (blue line) evolution; (**b**) kinetics of particles size (blue line) and polydispersity (red line) evolution over time. Data are related to test 3 in Table 1. * The highest data of ethyl acetate residua expressed in ppm are only indicative.

In order to better understand the emulsion preparation process, different samples were singled out at distinct times during the evaporation of the EtOAc and they were analyzed through DLS and head-space GC. Furthermore, the evolution of the dry matter content, while EtOAc evaporates, was followed. The experiment was carried out under the condition of entry 3 in Table 1. The evolution of the dry matter content shows an almost linear increase over time (Figure 2a). On the contrary, the EtOAc residue initially decreases slowly, and then almost exponentially. The difference among the two kinetics clearly indicates that during the evaporation stage not only the organic solvent but also a portion of water is removed from the preparation. This is due to the minimum temperature of the azeotrope between water and EtOAc.

In the initial period (0–3 h) when the EtOAc residuum is very high (~40%) but also later on, when it is lower, e.g., 37% at 5 h, the preparation is unstable and coagulates by standing. On the contrary, when the EtOAc residuum reaches the value of 80 ppm, after 19 h, the preparation is almost stable; the DLS correlogram exhibits just a slight decrease of the intensity during the analysis time and the emulsion was visibly stable. Besides, when the EtOAc residuum is only 33 ppm (after 22 h), the dispersion is very stable, even after a month of storing in the fridge at 4 °C (Figure 1). Overall, the data collected show that, in order to ensure the stability of the dispersion, the organic solvent must be completely removed (residue of EtOAc lower than 40 ppm).

During EtOAc evaporation, particle size and polydispersity evolve, both passing through a maximum after 90–100 min and then decreasing to reach the minimum after 1 day (Figure 2b). The initial increase of size can be due to coalescence between distinct particles. Indeed, after preparation, the dispersed phase is made of drops of PLA solution in EtOAc. As the solvent evaporates, the particle volume decreases and then also its size. Furthermore, due to the evaporation of EtOAc, the concentration of PLA in the particle increases and therefore, the viscosity of the oil phase increases also. As a result, the rate of coalescence between distinct particles decreases and becomes irrelevant once particles are solid.

### 3.3. Effect of Stabilizer on Dispersions Stability

With the purpose of studying the role of starch on the stability of the emulsion/dispersion, different trials to prepare the emulsion with only SDS as surfactant or with other polysaccharides than starch were performed (Table 2). Two different tests were carried out with only SDS as surfactant. The water phase amount, composition and the total amount of PLA were the same. The only difference between the two tests was the concentration of the oil phase, which was 11% and 8%, (entries 1 and 2, Table 2, respectively), and as consequence the oil phase amount and the oil/water phase ratio also changed. In both cases, PLA dispersions unstable by DLS analysis were afforded. The comparison with the high stability of the dispersions prepared under comparable conditions with the additional presence of starch (entry 3 and 5 in Table 1, respectively) clearly indicates that the presence of such agent is of utmost importance for the stabilization of the resulting formulations. Usually, starch can act as an emulsifier only if hydrophobic modifications are introduced on its chain in order to afford substantial surface activity at the oil-water interface [75]. Gelatinized starch, as a non-surface-active polysaccharide, has been reported to act as stabilizer for the emulsion by forming a secondary steric stabilization layer through interaction with the pre-adsorber emulsifier [65,68].

After the result with starch, we decided to explore if similar results could be obtained with other polysaccharides. To this aim, microfibrillated cellulose and xanthan gum were tested. Microfibrillated cellulose (MFC) in water has been reported to form a three-dimensional network capable of incorporating dispersed oil droplets, preventing their coagulation and sedimentation [76]. On the other hand, xanthan gum (XG) is widely used to increase the viscosity of the aqueous phase, hence preventing creaming of oil droplets [77]. However, in the case of the PLA, the attempt to employ MFC as stabilizer in a concentration of 0.5 wt.% in water, in the presence of SDS, gave a PLA dispersion that sedimented over time (entry 3, Table 2). On the other hand, any attempt to prepare PLA dispersions in the presence of higher MFC content was not successful because the mixtures could not be homogenized under ultrasound treatment due to the extremely high viscosity of the water phase.

Similar viscosity constraints were also observed with XG. Indeed, tests with XG as stabilizer provided stable dispersions only at XG concentration in water of 0.009 wt.% (entry 5, Table 2). The high viscosity of more concentrated XG solution, even 0.1 wt.%, prevented the effective emulsification of oil-and-water phases. Dispersion with 0.009 wt.% concentration of XG has nanoparticles with 192 nm diameter and dry residual of 14%. Both values are comparable to the ones afforded with starch as stabilizer, thus suggesting comparable mechanisms of stabilization for the two polysaccharides. Unluckily, when the preparation was performed at a larger scale (3 times higher) with XG, coagulation occurred (entry 6, Table 2). Overall, a PLA dispersion in water is formed in all the conditions listed in Table 2. However, only with starch as stabilizer is the formulation stable over time and it maintains its stability even when it is prepared at a higher scale, thus highlighting the key role of this polysaccharide in efficiently stabilizing the preparations, without preventing the effective homogenization of phases during the emulsion preparation.

Furthermore, the good stability of dispersions with starch, in spite of the lower viscosity of starch solutions with respect to XG, suggests that the viscosity is not the parameter that controls the dispersion stability.

### 3.4. Screening of Different Emulsifiers for the Formation and Stability of the Dispersion

In order to study the role of the emulsion stabilizer used in the formation and stability of the preparations, several surfactants in different combinations and concentrations were tested (Table 3).

At first, SDS in combination with Span 80, SYN (a PEO-PPO block copolymer), alone and in combination with Span 80 and Tween 80 were tested in the absence of starch (from entry 2 to 9, Table 3). However, only SDS in combination with Span 80 (entry 2, Table 3) and SYN at 2.4% concentration (entry 4, Table 3) provided stable emulsions. In the case of SYN, an almost satisfactory emulsion was already obtained at 0.8% (entry 6, Table 3). However, PLA coagulated on the stirrer forming a clot during the subsequent solvent evaporation, when the concentration was other than 2.4% (entries 3, 5, 6 and 7, Table 3). The dispersion obtained with SYN possesses a particle size between 400 and 450 nm. The value is higher than the one obtained with SDS. On the contrary, the particle size observed with SDS and Span 80 was around 150 nm, which is comparable to the one with only SDS.

Met. 388, SA, NaCMC, SSL and Met. 368 (entries 12, 15, 16, 17 and 18, Table 3) tested in the presence of starch, all led to the formation of inverted emulsions (water in oil instead of oil in water). On the other hand, with the use of Tween 80 and Tween 20 (entries 9, 13 and 14, Table 3) all the preparations were unstable due to coagulation.

**Figure 3:**
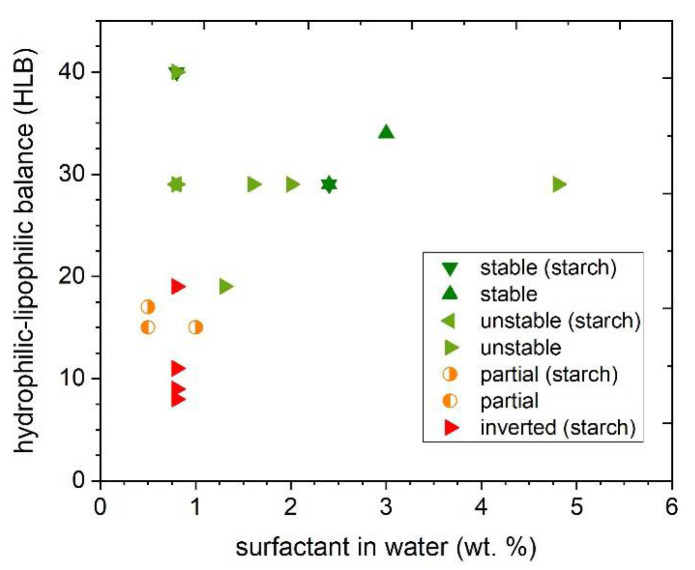
Influence of hydrophilic–lipophilic balance (HLB) and concentration of surfactant in water on the formation and stability of the formulations.

**Figure 4:**
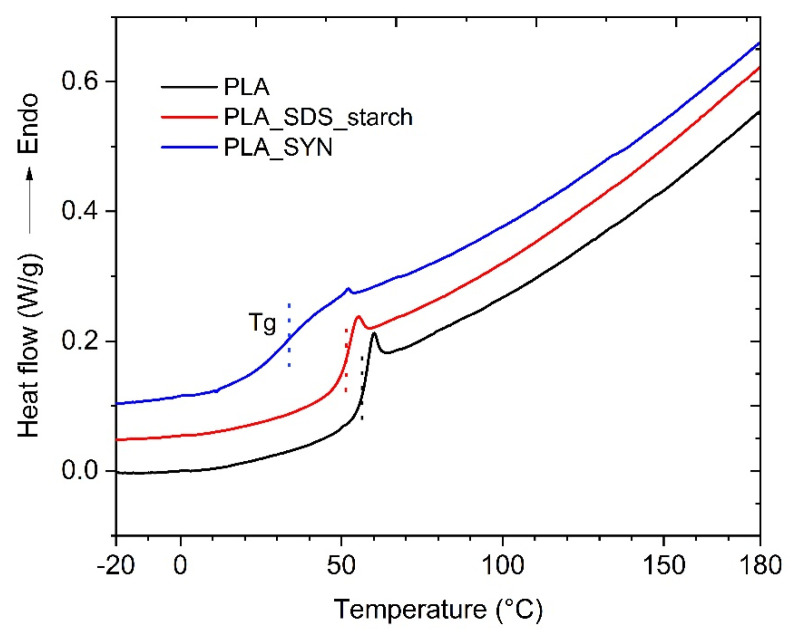
DSC thermograms at 10 °C/min of neat PLA, dried PLA dispersion with SDS and starch as surfactant and stabilizer, respectively, (entry 3, Table 1) and dried PLA dispersion with SYN (entry 23, Table 3). Second heating step. Thermograms were arbitrarily vertically shifted for clarity.

In order to rationalize the obtained results, they are visualized in Figure 3: the formation and stability of the PLA preparations are plotted as a function of the surfactants’ hydrophilic–lipophilic balance (HLB) values and their concentration in the water phase. The data highlight that for relatively low values of HLB (between 5 and 11) and low concentration in water (from 0.5 to 0.1 wt.%), an inverted emulsion (water-in-oil) is formed (red symbols, Figure 3). When the hydrophilic–lipophilic balance (HLB) value is in the 15–20 range, without exceeding concentration values of 0.1 wt.% in water, the emulsion sometimes is still inverted and sometimes partially forms (respectively, red and orange symbols, Figure 3). However, in this HLB range it is sufficient to increase the concentration of surfactant from 0.8 to 1.3 wt.% to obtain a direct emulsion (oil-in-water) (light green symbols, Figure 4). However, emulsions are not stable over time. Indeed, for hydrophilic–lipophilic balance (HLB) values higher than 19 and a concentration of surfactant higher than 1 wt.%, a direct emulsion was obtained with all tested surfactants, even if it is stable only in a few cases. In summary, two stability zones can be highlighted from the chart (dark green symbols, Figure 3): (a) for a high HLB value (40), a relatively low concentrations of surfactant (0.8 wt.%) and starch as stabilizer; and (b) with modest HLB (29 and 34) and fairly high concentrations of emulsifier (2 and 3 wt.%). In the second case, the use of starch as stabilizer does not affect the stability of the preparations, suggesting that, in a certain range of HLB values and concentrations of emulsifier, no stabilizing agents are needed. As a drawback, the use of a higher amount of surfactant significantly affects the composition of the final coating, therefore most likely also the final properties of the layer.

### 3.5. Characterization of the Dispersions

The dried dispersions were characterized through gel permeation chromatography (GPC) analysis in order to verify if the PLA was subjected to degradation during the preparation of the formulation and the subsequent storage. The comparison between the molecular weight of pristine PLA and PLA of dispersions showed minor differences (3–5%) in the range of the experimental error (Table 4). Furthermore, no significant change in polymer dispersity was detected, thus indicating the absence of significant degradation of the polyester during the processing in water for the dispersion preparation under ultrasound treatment and moderate heating and the subsequent storage for a few months.

DSC analysis of neat PLA 4060D showed glass transition temperature at 54.1 °C and no melting or crystallization peaks (Figure 4), in agreement with the amorphous nature of the resin declared by the producer (NatureWork processing guide NWPG002_020111; https://doi.org/10.1016/j.reactfunctpolym.2017.06.013, accessed on 1 September 2020). The analysis of dried dispersion with SDS and starch (entry 3, Table 1) showed only a modest shift of the Tg value towards a lower value (Tg = 51 °C) with respect to the pristine polyester, thus indicating a modest effect of the emulsion process even on the thermal properties of the resin. On the contrary, dispersion with SYN showed a significant decrease of ~20 °C in the glass transition temperature (Tg = 34 °C), indicating a plasticization of PLA by the PEO block of SYN [78]. This latter sample, in addition to the shift in the Tg’s mean value, showed a broadening of the transition, thus reflecting the variety of thermal motions available [79]. This last effect can be due to a non-homogeneous distribution of SYN in the PLA particles or even to a non-homogeneous distribution of SYN among particles, resulting in particles richer in the block copolymer and others with comparable depletion.

### 3.6. Preparation of Films from PLA Dispersions

**Figure 5:**
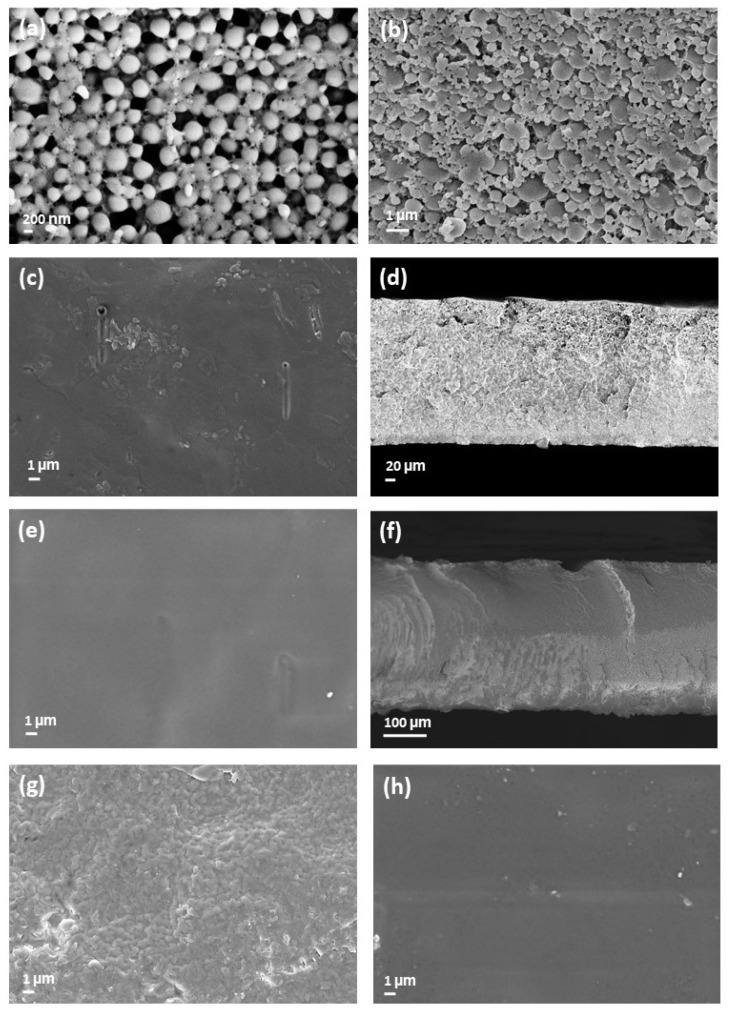
SEM pictures of the filming tests performed with water dispersions of PLA at room temperature (**a**,**b**), at 60 °C (**c**–**f**) and at 80 °C (**g**,**h**). Picture (**a**,**c**,**d**,**g**) refer to tests with the SDS_starch formulation; (**b**,**e**,**f**,**h**), correspond to tests with the SYN formulation.

**Figure 6:**
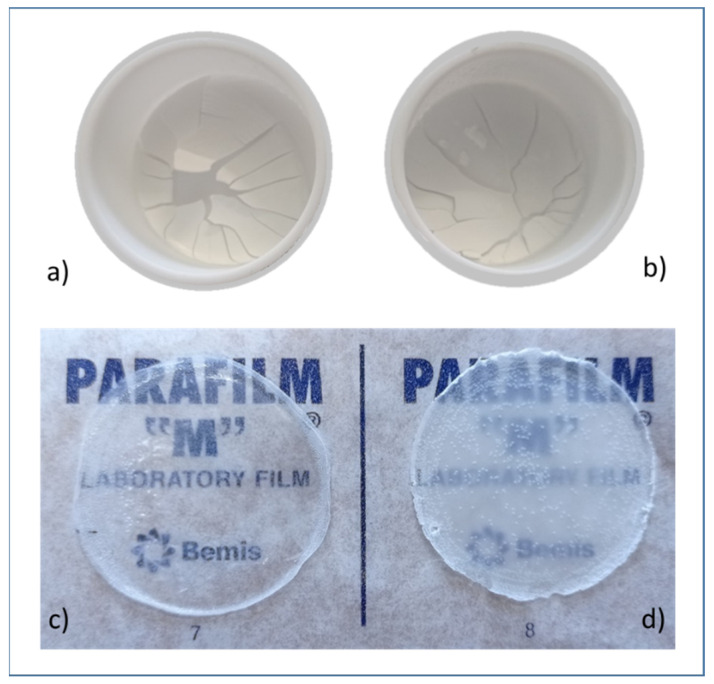
Pictures of PLA films obtained by solution casting at 40 °C (**a**,**b**) and 60 °C (**c**,**d**) of water dispersions with SDS and starch (**a**,**d**) or SYN (**b**,**c**).

PLA dispersions in water prepared with either SDS_starch (entry 3, Table 1) or SYN (entry 29, Table 3) as surfactant and stabilizer were cast on PTFE capsules and left to dry. When drying was performed at room temperature, a layer of powder was obtained. SEM analysis revealed the presence of submicrometric particles (Figure 5a,b), whose dimension was comparable to the values by DLS data of the corresponding water dispersion (Section 3.2). This result indicates that particles did not merge, most likely because the processing temperature was lower than the glass transition value of both dispersions (Section 3.5). When drying was performed at 40 °C, particles started to merge, forming white continuous layers. However, these were brittle and fractured (Figure 6a,b). A further increase of the temperature to 50 °C had a partial positive effect only in the case of the SYN dispersion, which has a glass transition temperature of 34 °C. However, the formed film, even if partially transparent, was brittle and cracked. On the contrary, when films are dried at 60 °C, continuous free-standing films with thickness between 230 and 550 µm are afforded (Figure 6c,d) with both formulations.

The formulation with SYN gave even, almost transparent films, while in the presence of SDS and starch, opaque films are afforded. In both cases, individual particles cannot be distinguished by SEM observation of the film’s surfaces (Figure 5c,e,g,h) and section (Figure 5d,f not shown), thus indicating effective film formation. Notice that 60 °C is higher than the glass transition temperature of both dispersions and of the pristine PLA. Processing at higher temperatures, up to 110 °C, provided films that look like the one obtained at 60 °C in the case of the SYN formulation. However, with the dispersions with SDS and starch, yellowing occurred at drying temperatures above 90 °C. Indeed, the SEM analysis of PLA dispersion in water prepared with SDS_starch at 80 °C (Figure 5g) shows the presence of granules that are probably due to the degradation of the surfactant/stabilizer.

Transmission measurements showed that PLA films with SDS and starch (thickness of 320 µm) possess a transmittance of 2.6% at 600 nm. The low transmittance is due to the whitish color of the film, probably given by the presence of starch. On the other hand, PLA film with SYN (thickness of 250 µm) showed a higher transparency (transmittance of 40% at 600 nm). However, the latter possesses a modest transparency considering that a pure PLA film prepared from EtOAc (thickness of 130 µm) has a transmittance of 72% at 600 nm.

**Figure 7:**
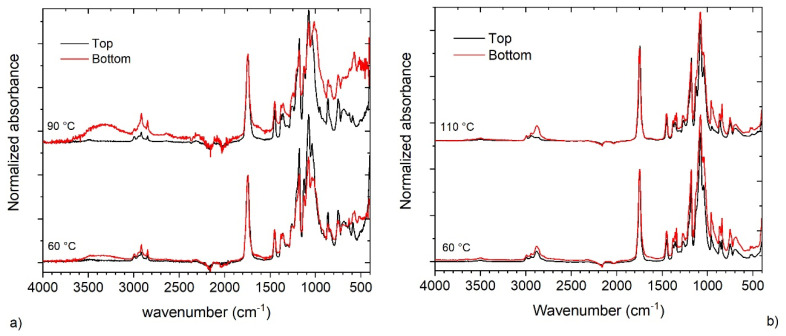
(**a**) FT-IR spectra of the top and bottom surfaces of PLA_SDS_starch films prepared at 60 and 90 °C; (**b**) FT-IR spectra of the top and bottom surfaces of PLA_SYN film prepared at 60 and 110 °C.

ATR analysis of the upper and lower surfaces of the films showed comparable band intensity, hence indicating a similar composition. As a consequence, significant surfactant segregation or blooming effects can be excluded (Figure 7). However, films obtained at high drying temperatures (90 and 110 °C) showed a different composition between the upper and lower surface by ATR analysis (Figure 7). In particular, an enrichment in stabilizer/surfactant is observed on the lower surface of the films. Indeed, the IR spectra of the bottom surface of PLA_SDS_starch film prepared at 90 °C (Figure 7a) shows a broad band between 3000 and 3500 cm^−1^, which is typical of the starch’s OH stretching. The enrichment of starch is further confirmed by the typical stretching band of sugar rings at around 1000 cm^−1^. Similarly, the film from the PLA_SYN formulation obtained at 110 °C, in the bottom surface showed more intense signal than the top surface. These are signals at nearly 2800 cm^−1^ relative to CH_2_ stretching and the one at nearly 955 cm^−1^, which are both present in the ATR spectra of Synperonic.

The possible degradation of PLA during film formation was checked by GPC analysis (Table 4). A modest variation, even if detectable (10–15% decrement), was observed after drying at 60 °C. Higher processing temperature resulted in a more extended molecular weight decrement. No variation of the dispersity was detected as a result of a comparable decrement in both the $\bar{M_{n}}$ and $\bar{M_{w}}$ value, thus suggesting no preferential degradation of the high or low molecular weight fractions. In any case, no optimization of the drying time has been performed and it can be envisaged that better control over the molecular weight can be achieved by proper control of the heating time.

## 4. Conclusions

In the present work, a procedure based on emulsification-evaporation method, which gives PLA dispersions in water with dry matter content up to 20 wt.%. has been developed. The procedure is based on the combination of an amorphous commercial PLA grade, ethyl acetate as solvent and surfactants all approved for food contact applications, so that the new preparation is also suitable for this application’s purpose.

Surfactants with hydrophilic–lipophilic balance (HLB) in the 29–34 range at 2 and 3 wt.% concentration are needed to stabilize the formulations during the preparation stages. Surfactants with higher HLB (40) are also effective if starch is included as stabilizer. Emulsification needs high energy input by ultrasound sonication to achieve effective dispersion. Furthermore, the total treated volumes, the organic/water volume ratio and the viscosity of phases must be adjusted to the sonication capability to achieve the stability of the final dispersion. In addition, for long-term stability, the final dispersion formulation must contain negligible ethyl acetate residua (<40 ppm). Under this circumstance, dispersions do not sedimentate or cream and particle size does not appreciably change over several months at room temperature or in the fridge at 4 °C.

The obtained dispersions, consisting of submicrometric PLA particles, were successfully cast into films, with minimum formation temperature of 60 °C, which is a little above the glass transition temperature of the pristine PLA.

## 5. Patents

The method of preparing halogen-free aqueous dispersion of biodegradable polymers was submitted to the Italian Patent Office (Bertoldo, M.; Ricci, L.; Messina, T.; Belletti, G.; Gallur Blanca, M.; Guillem Ortiz, A. and Aragón Gutierrez, A. Italian Patent issue no. 102020000028640, 26 November 2020).

## Figures and Tables

**Table 1:** Experimental conditions adopted to prepare ethyl acetate-(EtOAc) in-water PLA dispersions with SDS as surfactant and starch as stabilizer.

Entry	Organic Phase		EtOAc/H_2_O	SDS Conc. (wt.%)	Vol.Tot. (mL)	PLA/Water ^2^ (wt.%)	Dispersion Features		
	PLA (wt./vol.%) ^1^	Vol. (mL)	Vol. Ratio				Homogenizer ^3^	Stability ^4^	Particles Size ^5^ (nm)
1	11	45.0	1.3	0.8	80	14	UT	coa.	-
2	11	45.0	1.3	0.8	80	14	UH	yes	214 ± 2
3	11	45.0	1.3	0.8	80	14	UVC	yes	203 ± 7
4	11	62.5	1.8	0.8	97	20	UVC	cl.	225 ± 3
5	8	62.5	1.8	0.8	97	14	UVC	yes	178 ± 2
6	8	20.8	1.8	0.8	32	14	UVC	yes	167 ± 1
7	8	87.5	2.5	0.8	122	20	UVC ^6^	cl.	202 ± 2
8	8	87.5	2.5	0.8	122	20	UH ^6^	yes	130 ± 10
9	8	50.0	2.5	0.8	70	20	UVC	coa.	-
10	8	50.0	2.5	0.8	70	20	UH ^6^	yes	138 ± 23
11	8	50.0	5.0	0.8	60	15	UH	coa.	-
12	8	50.0	2.1	0.3	74	17	UH	yes	158 ± 12
13	8	20.8	1.8	0.5	58	15	UVC	yes	183 ± 2

^1^ Ratio between the weight of poly(lactic acid) and the volume of EtOAc. ^2^ Ratio between the weight of poly(lactic acid) and the volume of water. ^3^ UH = ultrasonication with Hielscher, UVC = ultrasonication with Vibracell, UT = Ultraturrax. ^4^ Assessed through visual criteria and DLS analysis both after the preparation and after 1 week. Stability as follows: coa. = coagulation, cl. = clot formation. ^5^ Determined through DLS analysis. ^6^ 1.1 wt.% of starch in water was employed.

**Table 2:** Screening of different stabilizers for the preparation of PLA dispersions in the presence of SDS as surfactant.

	Aqueous Phase		Organic Phase		EtOAc/H_2_O	Dispersion Feature
Entry	Stabilizer	wt.%	PLA (wt./vol.%) ^1^	Vol. (mL)	Vol. Ratio	Stability ^2^
1	-	-	11	45.0	1.3	no
2	-	-	8	62.5	1.8	no
3	MFC	0.500	11	45.0	1.3	sed.
4	XG	0.090	11	15.0	1.3	no
5	XG	0.009	11	15.0	1.3	yes
6	XG	0.009	11	45.0	1.3	coa.

^1^ Ratio between the weight of poly(lactic acid) and the volume of EtOAc. ^2^ Assessed through visual criteria and DLS analysis both after the preparation and after 1 week. Stability as follows: coa. = coagulation, sed. = sedimentation.

**Table 3:** Screening of different surfactants for the preparation of PLA dispersions.

Entry	Surfactant	Description	Aqueous Phase			Dispersion Stability ^1^
			Surfactant Conc. (wt.%)	HLB	Starch Conc. (wt.%)	
1	SDS	Anionic surfactant	0.8	40	0	unstable
2 ^2^	SDS Span 80	Anionic surfactant Non-ionic surfactant	2.5 0.5	34	0	stable
3	SYN	Block copolymer PEO/PPO	4.8	29	0	unstable
4	SYN		2.4	29	0	stable
5	SYN		2.0	29	0	unstable
6	SYN		1.6	29	0	unstable
7	SYN		0.8	29	0	unstable
8	SYN Span 80	Block copolymer PEO/PPO Non-ionic surfactant	0.8 0.5	19	0	unstable
9	Tween 80	Non-ionic surfactant	0.5	15	0	partial
10	SYN	Block copolymer PEO/PPO	2.4	29	0.5	stable
11	SYN		0.8	29	0.5	unstable
12	Met. 388	Non-ionic compound	0.8	19	0.5	inverted
13	Tween 20	Non-ionic surfactant	0.5	17	0.5	partial
14	Tween 80	Non-ionic surfactant	1.0	15	0.5	partial
15	SA	Anionic polysaccharide	0.8	11	0.5	inverted
16	NaCMC	Anionic cellulose derivative	0.8	11	0.5	inverted
17	SSL	Anionic surfactant	0.8	9	0.5	inverted
18	Met. 368	Ester based	0.8	8	0.5	inverted

^1^ Assessed through visual criteria and DLS analysis both after the preparation and after 1 week. Inverted = formation of water-in-oil emulsion instead of oil-in-water one. ^2^ Homogenization with UH, PLA/EtOAc ratio (wt./vol.%) = 8, EtOAc/H_2_O vol. ratio = 2.5, total volume = 70 mL.

**Table 4:** Molecular weight by GPC analysis of pristine PLA and PLA dispersions dried at different temperatures.

Sample	Drying Temperature (°C)	$\bar{M_{n}}\;(\mathrm{kDa})$	$\bar{M_{w}}\;(\mathrm{kDa})$	Đ
PLA	-	113.8	166.1	1.46
PLA_SDS_starch	rt	109.6	157.8	1.44
PLA_SYN	rt	119.5	164.9	1.38
PLA_SDS_starch	60	96.8	141.3	1.46
PLA_SYN	60	102.2	134.9	1.32
PLA_SDS_starch	80	92.3	133.8	1.45
PLA_SYN	80	85.6	123.3	1.44
PLA_SDS_starch	100	99.6	143.4	1.44
PLA_SYN	100	88.9	131.5	1.48

## Data Availability

The data presented in this study are available on request from the corresponding author.
